# Role of Prohibitins in Aging and Therapeutic Potential Against Age-Related Diseases

**DOI:** 10.3389/fgene.2021.714228

**Published:** 2021-10-29

**Authors:** Misa Belser, David W. Walker

**Affiliations:** ^1^ Department of Molecular, Cell, and Developmental Biology, University of California, Los Angeles, Los Angeles, CA, United States; ^2^ Department of Integrative Biology and Physiology, University of California, Los Angeles, Los Angeles, CA, United States; ^3^ Molecular Biology Institute, University of California, Los Angeles, Los Angeles, CA, United States

**Keywords:** prohibitin, aging, age-related diseases, PHB1, PHB2

## Abstract

A decline in mitochondrial function has long been associated with age-related health decline. Several lines of evidence suggest that interventions that stimulate mitochondrial autophagy (mitophagy) can slow aging and prolong healthy lifespan. Prohibitins (PHB1 and PHB2) assemble at the mitochondrial inner membrane and are critical for mitochondrial homeostasis. In addition, prohibitins (PHBs) have diverse roles in cell and organismal biology. Here, we will discuss the role of PHBs in mitophagy, oxidative phosphorylation, cellular senescence, and apoptosis. We will also discuss the role of PHBs in modulating lifespan. In addition, we will review the links between PHBs and diseases of aging. Finally, we will discuss the emerging concept that PHBs may represent an attractive therapeutic target to counteract aging and age-onset disease.

## Introduction

There are two prohibitin subunits, prohibitin 1 (PHB1) and prohibitin 2 (PHB2), which together form a ring structured heterodimeric complex about 20–25 nm in diameter (Artal-Sanz and Tavernarakis, 2009a). PHBs are evolutionarily conserved and ubiquitously expressed in many types of tissues. PHB1 weighs 32 kDa and PHB2 weighs 34 kDa (Artal-Sanz and Tavernarakis, 2009a). Although PHBs are largely found in the inner mitochondrial membrane, they have also been found in the nucleus, cytosol, plasma membrane, endoplasmic reticulum, and macrophage phagosomes (Garin et al., 2001; Signorile et al., 2019).

PHBs were named “prohibitins” because they were initially found to prohibit initiation of DNA synthesis (McClung et al., 1989). PHBs have a diverse range of functions associated with aging, such as apoptosis, cellular senescence, cancer, and mitochondrial metabolism (Artal-Sanz and Tavernarakis, 2009a). PHBs also assist with protein folding of complexes in the electron transport chain (Nijtmans et al., 2002). PHB1 and PHB2 form a complex which acts as a chaperone by binding to products of mitochondrial translation and preventing their degradation by metalloproteases (Nijtmans et al., 2002). PHB2 acts as a protein-lipid scaffold (Artal-Sanz and Tavernarakis, 2009a). Both subunits of the PHB complex have N-terminal domains to anchor them in the inner mitochondrial membrane (Artal-Sanz and Tavernarakis, 2009a).

## Prohibitins and Mitophagy

Mitophagy is the breakdown of damaged mitochondria via autophagy (Pickles et al., 2018). Mitophagy and mitochondrial dynamics (fission/fusion) are linked in maintaining mitochondrial quality control (Youle and van der Bliek, 2012). Excessive mitochondrial fusion/impaired mitochondrial fission is an underlying factor in the age-related decline in mitophagy, which is associated with senescence and aging (Rana et al., 2017; Aparicio et al., 2019; D'Amico et al., 2019). PHB2 binds to microtubule-associated protein 1A/1B-light chain 3 (LC3) to promote degradation of the mitochondria by an autophagosome (Tanida et al., 2008; Wei et al., 2017). Through oligomycin + antimycin (OA)-induced mitophagy, it was found that the mitochondrial membrane must be ruptured before PHB2 can bind to LC3 (Wei et al., 2017).

When there is a loss of mitochondrial membrane potential, PTEN-induced kinase 1 (PINK1) is unable to be degraded by presenilins-associated rhomboid-like protein (PARL), accumulates on the outer mitochondrial membrane, and recruits Parkin to mediate mitophagy (Jin et al., 2010). Parkin then ubiquitinates the mitochondrial fusion-promoting factor Mitofusin (Mfn) (Poole et al., 2010; Tanaka et al., 2010; Ziviani et al., 2010) and other mitochondrial proteins (Chan et al., 2011; Wang et al., 2011), promoting the segregation and autophagic turnover of the dysfunctional mitochondria (Youle and Narendra, 2011; Ashrafi and Schwarz, 2013).

A second study found further support that PHBs can promote mitophagy. This study found a new axis for mitophagy by PHB2: PHB2-PARL-PGAM5-PINK1 (Yan et al., 2020). Loss of PHB2 prevented mitophagy by destabilizing PINK1, which inhibited recruitment of Parkin, optineurin, and ubiquitin. Conversely, increasing PHB2 levels was found to increase mitophagy by promoting Parkin recruitment. Additionally, this study discovered that a synthesized ligand for PHBs, FL3, suppressed cancer by inhibition of mitophagy by PHB2. FL3 is a flavagline that has been shown to have a cardioprotective effect (Qureshi et al., 2015).

## Prohibitins and Cellular Senescence

It is known that after a certain number of divisions, cells become senescent and stop dividing (Campisi and d'Adda di Fagagna, 2007). The number of senescent cells increases exponentially with age (He and Sharpless, 2017). As a result, a better understanding of the aging process may result from studying alterations in senescent cells. Levels of PHB2 decrease in senescing cells. The amount of mRNA coding for both PHB1 and PHB2 also decreases for yeast cells undergoing senescence (Piper et al., 2002). In addition, PHB1 and PHB2 levels decline for CEF chick embryo fibroblasts and HF19 human fibroblasts undergoing senescence (Coates et al., 2001). However, neither of these studies conclude that the decline in PHBs during senescence directly affects aging.

An early study on PHBs compared PHB1 levels in human cells between cells with low population doubling level (PDL) and cells with high PDL (Liu et al., 1994). PHB1 mRNA and protein levels were similar between low PDL cells and high PDL cells. However, a Western blot detected two PHB1 isoforms for low PDL cells, but only one PHB1 isoform for high PDL cells. This finding suggests that PHB1 in low PDL cells is post-translationally modified, but PHB1 in high PDL cells is not (Liu et al., 1994). The conclusion of Liu et al. (1994) that PHB1 mRNA and protein levels remain the same is also inconsistent with the results of Coates et al. (2001) and Piper et al. (2002).

PHB1 induces senescence in cells by synergizing with heterochromatin protein 1γ (HP1γ) to reduce transcription facilitated by E2F1 (Rastogi et al., 2006a). E2F1 is a transcription factor that acts as a regulator of promotors involved in cell division (DeGregori et al., 1995; Rastogi et al., 2006a). PHB1 recruits HP1γ to E2F1-controlled promoters to repress them. This recruitment was observed in senescent cells but not quiescent cells (Rastogi et al., 2006a). In addition, the study found that PHB1 depletion resulted in a reduced senescent phenotype.

Petunia flowers with silenced *PhPHB1* senesce faster than unsilenced flowers (Chen et al., 2005). This finding suggests that *PhPHB1* modulates the beginning of cellular senescence in flora. Silenced flowers also underwent fewer cellular divisions, with their petals containing only about 15% of the number of cells in the petals of the control group (Chen et al., 2005). Flowers with silenced *PhPHB1* also had higher respiration rates and higher levels of catalase transcripts, which help protect cells from reactive oxygen species (ROS) (Chen et al., 2005). This finding suggests that there were higher ROS levels for the flowers with silenced *PhPHB1*. In endothelial cells, ROS have been shown through knockdown of PHB1 to lead to senescence (Schleicher et al., 2008).

## Role of Prohibitins in Modulating Lifespan

*PHB1* and *PHB2* are the genes that encode the prohibitin proteins in yeast, Phb1p and Phb2p (Coates et al., 2001). Levels of Phb1p appear to be contingent on the levels of Phb2p in *Saccharomyces cerevisiae*. Deletion of *PHB1* leads to a lack of Phb2p and deletion of *PHB2* results in an absence of Phb1p (Berger and Yaffe, 1998). As a result, it was thought that depletion of either Phb1p or Phb2p would have the same effect on lifespan as the depletion of both Phb1p and Phb2p. However, it was discovered that the loss of both Phb1p and Phb2p had a greater effect on replicative lifespan than the loss of either alone (Piper et al., 2002). This finding was consistent with the results of Coates et al. (1997) but was inconsistent with Berger and Yaffe’s (1998) study, which found that the *phb1*-null *phb2*-null mutation had a less severe effect on replicative lifespan than the *phb1*-null mutation in MYY290 or MYY291 yeast cells with wild-type mitochondrial genomes.

There are different models of aging in yeast, which include replicative lifespan and chronological lifespan (Longo et al., 2012). Replicative lifespan measures the number of times a cell can divide, while chronological lifespan measures how long a non-dividing yeast cell stays alive (Longo et al., 2012). Deletion of PHB1 does not affect the chronological lifespan of *S. cerevisiae* cells stuck in G0 (Artal-Sanz and Tavernarakis, 2010). Piper et al. (2002) discovered that the chronological lifespan of yeast was relatively unchanged after the loss of Phb1p and Phb2p.

There are conflicting findings regarding the effect of PHB1 gene deletion on replicative lifespan in *S. cerevisiae*. One study observed that deletion of the PHB1 encoding gene in yeast haploid cells increases replicative lifespan by approximately 30%, while overexpression of the gene results in about a 20% decrease in lifespan (Franklin and Jazwinski, 1993). The deletion of the gene was thought to increase lifespan by allowing cells to divide for longer than they normally would have. In contrast, another study discovered that deletion of either *PHB1* or *PHB2* reduces the replicative lifespan in *S. cerevisiae* and leads to changes typical in aging, such as a longer duration of cell division and larger cell size (Coates et al., 1997; Smith et al., 2015). Since both Franklin and Jazwinski (1993) and Coates et al. (1997) were examining replicative lifespan, it may be beneficial to examine the factors that contribute to different results when measuring replicative lifespan in yeast. It has also been noted that different effects on lifespan can be observed when measuring the replicative lifespan of yeast, depending on the type of strain and the growth medium used (Longo et al., 2012).

In *C. elegans*, the relationship between knockdown of PHBs and longevity is dependent upon genotype (Artal-Sanz and Tavernarakis, 2010). Either PHB1 or PHB2 knockdown with RNAi decreases lifespan of wild type worms (Artal-Sanz and Tavernarakis, 2009b). However, knockdown of PHB1 through RNAi has been shown to increase the lifespan of certain *C. elegans* mutants. Notably, *daf-2* mutants live 150% longer with either PHB1 or PHB2 knockdown (Artal-Sanz and Tavernarakis, 2009b). DAF-2 is a transmembrane insulin receptor kinase that modulates longevity in *C. elegans* (Kimura et al., 1997). Other *C. elegans* mutants found to live longer with knockdown of PHBs include electron transport chain mutants, mutants with changes in the metabolism of fat, and diet restriction mutants (Artal-Sanz and Tavernarakis, 2009b). In all these cases, knockdown of PHBs decreased intestinal fat and mitochondria levels. These findings suggest that although knockdown of PHBs decreases lifespan in wild type worms, knockdown of PHBs can increase lifespan in mutants with changed growth factor signaling, altered fat processing, or impaired mitochondrial performance (Artal-Sanz and Tavernarakis, 2010). As a result, the question of whether knockdown of PHBs increases or decreases lifespan in worms is dependent on the state of metabolism.

Although there are several studies examining the effects of depletion of PHBs on lifespan, experiments examining the effects of upregulation of PHBs on lifespan are notably lacking.

## Prohibitins and Oxidative Phosphorylation

PHBs affect enzymes involved in metabolic processes, including oxidative phosphorylation. PHB1 acts as an inhibitor for pyruvate carboxylase and allows for insulin-stimulated regulation of glucose and fatty acid oxidation (Vessal et al., 2006). This regulation by PHB1 downregulates oxidative phosphorylation and instead supports metabolism through anaerobic glycolysis, which may have a role in aging. PHB1 can be modified through phosphorylation, but fibroblasts become less able to phosphorylate PHB1 as the cells undergo senescence (Liu et al., 1994). Since PHB1 regulates oxidative phosphorylation, this change in modifications over time may be related to the 40% decrease in oxidative activity of mitochondria as people age (Petersen et al., 2003).

PHB1’s role in metabolism may have importance in cancer because its role as an inhibitor of pyruvate carboxylate facilitates the shifting from oxidative phosphorylation to anaerobic glycolysis (Vessal et al., 2006). PHB1 overexpression has been reported in cancer cells (Yang et al., 2018). This overexpression may help rapidly growing cancer favor anaerobic glycolysis over oxidative phosphorylation, to reduce the high oxidative stress that would occur if oxidative phosphorylation was used (Vessal et al., 2006).

There are changes in PHB1 localization in reaction to oxidative stress. A study found that under oxidative stress, PHB1 relocated from the mitochondria to the cell nucleus, where it can regulate transcription (Sripathi et al., 2011). However, another study found that PHB1 translocated from the nucleus to the mitochondria in the presence of the ROS H_2_O_2_ (Lee H. et al., 2010). Another study found that overexpression of PHB1 made neonatal rat cardiomyocytes less susceptible to apoptosis in response to oxidative stress by H_2_O_2_ (Liu, X. et al., 2009).

PHBs also support complexes involved in the electron transport chain. PHB2 can help facilitate normal mitochondrial respiration through its interaction with sphingosine-1-phosphate (S1P), a lipid mediator, by supporting the proper assembly of complex IV of the electron transport chain (Strub et al., 2011). PHBs may also help support complex I of the electron transport chain. A study found that PHB1 can protect complex I from the rotenone inhibitor and upregulation of PHB1 is connected to decreased manufacture of ROS (Zhou et al., 2012).

## Prohibitins in Age-Related Diseases

Degenerative diseases such as Alzheimer’s disease, Parkinson’s disease, diabetes, and cancer are often age-related. PHBs have been implicated in each of these diseases.

### Prohibitins and Alzheimer’s Disease

In a 2007 paper, researchers observed the frontal cortex of sporadic Alzheimer’s disease (AD) cases and did not observe changes in PHB1 levels (Ferrer et al., 2007). This finding was consistent with another paper published in 2008 that also examined the frontal cortex and found that levels of PHB1 for AD cases were similar to the levels in control cases (Pérez-Gracia et al., 2008). However, a later study in 2017 found a change in PHB1 levels when studying the olfactory bulb (OB) (Lachén-Montes et al., 2017). An early sign of AD is olfactory dysfunction (Zou et al., 2016). In intermediary and progressive AD phases, Lachén-Montes et al. (2017) observed PHB2 depletion. There were also lower levels of PHB1 isoforms that were phosphorylated. This result is consistent with observations that fibroblasts become less able to phosphorylate PHB1 with age (Liu et al., 1994).

Lachén-Montes et al. (2017) found that AD is associated with PHB2 depletion, which is consistent with another study that observed neurodegeneration and cognitive and behavioral disablements in neuron-specific PHB2-deficient (*Phb2* ^*NKO*^) mice (Merkwirth et al., 2012). Cognitive and behavioral disabilities in the mice were assessed using the Morris water maze paradigm, the elevated zero maze test, and open field tests. These impairments were associated with abnormal mitochondrial structure and hyperphosphorylated tau, a protein found mainly in neurons.

There is evidence that suggests the drug PDD005, which targets PHB1 and PHB2, can protect against neurodegenerative diseases such as AD and Parkinson’s disease (PD). PHB1 and PHB2 levels increase in the brains of aged mice when they are treated with PDD005 (Guyot et al., 2020). In contrast, the levels of the cytokine IL-1β decreased, suggesting that PDD005 and its interactions with PHB1 and PHB2 reduce inflammation in the brain. The authors discovered that PDD005 increases the expression of the signaling molecule GSK-3β, which can promote the destabilization of β-catenin through phosphorylation (Wu and Pan, 2010). This β-catenin interacts with nuclear factor-κ-light-chain-enhancer of activated B cell (NF-kβ) components to prevent transcription of proinflammatory molecules (Ma and Hottiger, 2016).

### Prohibitins and Parkinson’s Disease

There is not much research studying the relationship between PHBs and Parkinson’s disease (PD). It is known that dysfunctional mitochondria play a significant role in the development of PD and in preventing the death of neuron cells (Pickrell and Youle, 2015). PHBs are involved in mitochondrial control by promoting mitophagy to prevent the buildup of dysfunctional mitochondria observed in aging, suggesting that PHBs may protect against PD (Wei et al., 2017). This idea has been supported by a study on PD demonstrating that decreased levels of PHB1 increased the susceptibility of dopamine sensitive neurons to 1-methyl-4-phenyl-pyridnium (MPP+) instigated death while overexpression of PHB1 made them less susceptible (Dutta et al., 2018). MPP+ acts as a neurotoxin and is the oxidized product of 1-methyl-4-phenyl-1,2,3,6-tetrahydropyridine (MPTP) (Sian et al., 1999). MPTP administration has been shown to result in dopaminergic neuron degeneration (Muñoz-Manchado et al., 2016). However, MPTP has the limitation of not inducing some of the characteristics of PD (Sian et al., 1999).

There are also lower PHB1 levels in the substantia nigra, a brain structure known to be vulnerable in PD, with an estimated 60% loss of neurons in this region by the time of presentation of motor symptoms (Ferrer et al., 2007).

### Prohibitins and Diabetes

Another age-related disease that PHBs have been found to be implicated in is diabetes. Knockout of the gene for PHB2 in mouse pancreatic β-cells first leads to defective mitochondrial performance and defective secretion of insulin, then a decline in β-cells and continuing changes in glucose homeostasis, culminating in extreme diabetes (Supale et al., 2013).

Another study investigated the effects of PHB1 on diabetic cardiomyopathy. This study created a model of type 2 diabetes by feeding rats a diet high in fat while treating them with a low dose of streptozotocin, which is a toxin to pancreatic β-cells (Dong et al., 2016). PHB1 overexpression was achieved through lentiviral transduction. This transduction improved the deleterious effects in rats with diabetic cardiomyopathy, such as resistance to insulin, dysfunction of the left ventricle, fibrosis, and programmed cell death (Dong et al., 2016). However, the precise process in which PHB1 improves diabetic cardiomyopathy is not known.

Although increasing PHB1 levels helps reduce the effects of type 2 diabetes, it does not seem to be because PHB1 levels are lower in people with type 2 diabetes than in people who do not have type 2 diabetes. Levels of PHB1 in the serum of type 2 diabetic subjects are similar to the levels observed in control subjects (Kakarla et al., 2019).

### Prohibitins, Cancer, and Apoptosis

There have been many studies describing the relationship between PHBs and cancer (Koushyar et al., 2015). However, there is disagreement between studies about whether PHBs repress or support cancer and whether PHBs protect cancer cells from apoptosis or make them more susceptible.

High levels of PHBs are commonly observed in tumors. Coates et al. (2001) discovered that there are Myc oncoprotein binding sites in the promoters for PHB1 and PHB2 and that increasing Myc levels induces expression of PHB1 and PHB2. These findings suggest that levels of PHBs are often increased in tumors due to upregulation by oncoproteins.

Overexpression of PHB1 and PHB2 has been observed in blood-related cancers. PHB1 and PHB2 levels in lymphoma and leukemia cells were compared to healthy peripheral blood mononuclear cells (Ross et al., 2017). PHB1 and PHB2 levels were higher in these cancer patients than in controls. The overexpression of PHBs also protected these cancer cells from apoptosis induced by ROS. This finding suggests that cancer cells, with their higher use of glycolysis and therefore higher generation of ROS, can survive through the overexpression of PHBs. This finding that PHBs protect cancer cells from apoptosis was supported by another study in which silencing the expression of PHB1 made ovarian cancer cells more susceptible to apoptosis (Gregory-Bass et al., 2008).

Depletion of PHBs has been observed to make cells more receptive to apoptosis, while overexpression of PHBs makes cells less susceptible (Peng et al., 2015). For example, depletion of PHB1 in mice results in lethality during development and high levels of apoptosis (Park et al., 2005; He et al., 2008; Merkwirth et al., 2012; Peng et al., 2015). For the cell line Kit225, PHB1 and PHB2 knockdown with siRNA results in increased cell death through the ROS H_2_O_2_ (Ross et al., 2017).

Although there are studies showing that PHBs protect cells from apoptosis, there are a few studies that have found the opposite. The finding of Gregory-Bass et al. (2008) that silencing of PHB1 increased tumor cell susceptibility to apoptosis was contradicted by another study demonstrating that PHB1 knockdown results in inhibition of apoptosis in NB4-R1 leukemia cells (Liu et al., 2012). Rather than protecting cells from apoptosis, PHB1 overexpression was found to encourage apoptosis of cells in leukemia with arsenic sulfide treatment (He et al., 2015).

There are also conflicting findings regarding PHB1 levels in gastric cancer. In one study, researchers found that in gastric cancer, the microRNA miR-27a is upregulated, targets PHB1, and acts as an oncogene (Liu T. et al., 2009). Downregulating miR-27a in these gastric cancer cells increased levels of PHB1 protein and transcripts. Although the study by Liu T. et al. (2009) revealed that the levels of PHB1 in gastric cancer cells were lower than in healthy cells, another study found that they were higher (He et al., 2004).

There are also conflicting conclusions regarding the potential of PHBs as a treatment for cancer. Synthetic PHB1 mRNA can prevent DNA synthesis in HeLa cells and healthy fibroblasts (Nuell et al., 1991), providing evidence that PHB1 transcripts could be a potential treatment for cancer by preventing the replication of DNA in cancer cells. This finding that PHB1 transcripts can prevent DNA synthesis was supported by another study demonstrating that the PHB1 3′ untranslated region (3′UTR) encodes RNA that suppresses breast cancer by preventing entry into S phase of the cell cycle (Jupe et al., 2001). Further studies examining breast cancer revealed that PHB1 increases p53-regulated transcription while decreasing transcription regulated by E2F1 (Fusaro et al., 2003). As a result, these studies suggest that PHB1 could be used as a treatment for cancer by suppressing cancer cell proliferation and increasing transcription regulated by tumor suppressor proteins such as p53. Although Nuell et al. (1991) found that synthetic PHB1 mRNA microinjection suppresses proliferation of cancer cells, another study found that silencing PHB1 helps make drug-resistant cancer cells drug-sensitive (Patel et al., 2010). This study found that drug-resistant cancer cells tend to have higher levels of PHB1 on their surface compared to drug-sensitive cells.

These conflicting findings between studies may be due to the relationship between PHBs, cellular senescence, and apoptosis. PHBs can inhibit the proliferation of cancer cells by preventing their entry into S phase and inducing senescence (Nuell et al., 1991). This senescence could protect cancer cells from apoptosis. The location of PHBs may also affect whether they act as tumor supporters or suppressors. PHB1 on the plasma membrane is associated with drug-resistant cancer cells, while PHB1 in the nucleus is associated with tumor suppression (Patel et al., 2010; Theiss and Sitaraman, 2011). The location of PHBs may affect whether the cell will undergo apoptosis. Cells were protected from programmed cell death when PHB1 transport to the cytoplasm was inhibited (Rastogi et al., 2006b).

## Prohibitin as a Therapeutic Target

PHBs have been found to interact with a wide variety of molecules. Targeting the interactions between PHBs and these molecules may have therapeutic potential against age-related diseases. An outline of small molecules that interact with PHBs and their relevance to age-related diseases is provided in Table 1.

**TABLE 1:** Small molecules that interact with PHBs and their relevance to age-related diseases.

Molecule	Relevant subunits	Result of interaction	Relevant diseases	References
Aurilide	PHB1	Initiates apoptosis mediated by optic atrophy 1 (OPA-1)	Cancer	Sato et al., 2011;
		Induces mitochondrial fragmentation		Semenzato et al., 2011
		Interferes with the interaction between PHB1 and spastic paraplegia 7 (SPG7)		
Melanogenin	PHB1	Induction of pigmentation	Pigmentary disorders	Snyder et al., 2005;
		Can trigger apoptosis in tumor cells, such as melanoma cells	Cancers such as melanoma	Djehal et al., 2018
Mel9	Activates LC3, which interacts with PHB2	Promotes the melanocytic production of melanin	Pigmentary disorders	Djehal et al., 2018
		Mel9 (10 μM) induced apoptosis after a 48 h treatment in HBL, MM043, and MM162 cells	Cancers such as melanoma	
Mel41	Activates LC3, which interacts with PHB2	Promotes the melanocytic production of melanin	Pigmentary disorders	Djehal et al., 2018
		Mel41 (10 μM) induced apoptosis after a 48 h treatment in HBL, MM043, and MM162 cells	Cancers such as melanoma	
Mel55	PHB1	Mel55 (10 μM) induced apoptosis after a 48 h treatment in HBL and MM043 cells	Pigmentary disorders	Djehal et al., 2018
			Cancers such as melanoma	
Rocaglamide	PHB1 and PHB2	Prevents the interaction between PHBs and CRaf, leading to inhibition of the CRaf-MEK-ERK pathway	Cancer HCV	Polier et al., 2012; Luan et al., 2014;
		Prevents the entry of hepatitis C virus (HCV)		Liu et al., 2015;
		Changes PHB1’s localization to the plasma membrane		Wang et al., 2020
		Suppresses malignant cell proliferation, metastasis, cell growth and division, and protein synthesis		
		Inhibits mitophagy by blocking the interaction between PHBs and PARL		
FL3	PHB1 and PHB2	Inhibits mitophagy by PHB2	Cancer	Bernard et al., 2011;
		Reduces mortality by 50% in mice treated with doxorubicin	Protection against cardiotoxicity in cancer treatments	Qureshi et al., 2015;Wintachai et al., 2015;
		Protects cells from infection by chikungunya virus (CHIKV)	CHIKV	Yan et al., 2020
		Cardioprotection and STAT3 phosphorylation		
Fluorizoline	PHB1 and PHB2	Triggers apoptosis of chronic lymphocytic leukemia cells and acute myeloid leukemia (AML) cells	Cancer Pigmentary disorders	Pérez-Perarnau et al., 2014;
		Induces apoptosis by increasing levels of NOXA protein		Pomares et al., 2016;
		Triggers AML cells to differentiate and represses clonogenicity		Cosialls et al., 2017;
		Stops epidermal growth factor/RAS-induced CRaf activation		Yurugi et al., 2017;
		Modulates pigmentation in melanoma cells		Djehal et al., 2018
Spiro-oxindoles	PHB1 and PHB2	2′-phenylpyrrolidinyl-spirooxindole and its analogs protect against cytotoxicity from doxorubicin	Protection against cardiotoxicity in cancer treatments	Elderwish et al., 2020
		Support the survival of cardiomyocytes by leading to STAT3 phosphorylation		
Nitric Oxide (NO)	PHB1	PHB1 and NO are necessary for ischemic preconditioning, which increases the brain’s tolerance to ischemia, or reduced blood flow to the brain	Neurodegenerative diseases	Liu X. Q. et al. 2009;Qu et al., 2020
		NO directly regulates PHB1 through post-translational modification by protein S-nitrosylation		
PDD005	PHB1 and PHB2	Increases PHB1 and PHB2 levels in the brains of aged mice and decreases levels of the cytokine IL-1β	Alzheimer’s disease Parkinson’s disease	Guyot et al., 2020
Sulfonyl amidines	PHB1	PHB1 prevents the formation of osteoclasts	Osteoporosis	Lee M. Y. et al., 2010;
		Sulfonyl amidines limit resorption of bone and inhibit differentiation into osteoclasts		Chang et al., 2011; Lee et al., 2015;
		However, it is not known exactly how PHB1 affects sulfonyl amidines		
				Wang et al., 2020
Xanthohumol (XN)	PHB2	Prevents the growth of ERα positive breast cancer cells by disrupting the interaction between PHB2 and brefeldin A-inhibited guanine nucleotide-exchange protein 3 (BIG3)	Breast cancer	Yoshimaru et al., 2014
JI051	PHB2	Results in stabilization of the Hes family basic helix-loop-helix transcription factor 1 (Hes1) and PHB2 interaction, leading to cessation of the cell cycle in the G2/M phase	Cancer	Perron et al., 2018
		JI051 inhibits the proliferation of HEK293 cells		
JI130	PHB2	Results in stabilization of the Hes family basic helix-loop-helix transcription factor 1 (Hes1) and PHB2 interaction, leading to cessation of the cell cycle in the G2/M phase	Cancer	Perron et al., 2018
		JI130 decreases the volume of tumors		
Capsaicin	PHB2	Results in the translocation of PHB2 from the inner mitochondrial membrane to the nucleus	Cancer	Kuramori et al., 2009
		In the nucleus, PHB2 increases the transcriptional activity of p53, which induces apoptosis		
Adipotide	PHB1	Combats obesity and causes weight loss in Old World monkeys by resulting in apoptosis of the blood vessels of adipose tissue	ObesityInsulin resistance	Kolonin et al., 2004;Barnhart et al., 2011
		Adipotide also resulted in decreased insulin resistance		

### Prohibitins and Aurilide

The interactions of PHBs with small molecules can give insight into how PHBs modulate cellular processes such as apoptosis. Aurilide is a natural marine product from *Dolabella auricularia* and is cytotoxic (Suenaga et al., 2004). When PHB1 interacts with aurilide, it initiates apoptosis mediated by optic atrophy 1 (OPA-1) (Sato et al., 2011). The study by Sato et al. (2011) suggests aurilide can influence apoptosis through its interaction with PHB1. In contrast, PHB2 does not have affinity for aurilide (Sato et al., 2011; Semenzato et al., 2011).

Upregulating PHB1 makes cells less susceptible to aurilide (Sato et al., 2011). In contrast, partial downregulation of PHB1 through siRNA makes cells more susceptible. This finding suggests that aurilide interferes with the PHB complex. Sato et al. (2011) also discovered that aurilide induces mitochondrial fragmentation, which is similar to the mitochondrial morphology observed under PHB1 knockdown with siRNA.

Other studies revealed how aurilide’s interactions with PHB1 trigger apoptosis by affecting the morphology of mitochondria. PHB1 and spastic paraplegia 7 (SPG7) normally interact, but treatment with aurilide interferes with the interaction (Sato et al., 2011; Semenzato et al., 2011). SPG7 is part of the *m*-AAA protease which is involved in the processing of OPA-1 (Duvezin-Caubet et al., 2007). OPA-1 controls cristae dynamics, which involves a change in mitochondrial morphology (Griparic et al., 2004; Song et al., 2009).

OPA-1 is important for fusion of mitochondria (Cipolat et al., 2004; Song et al., 2009). Aurilide increased the formation of short (S) isoforms of OPA-1, which stop fusion and result in mitochondrial fragmentation (Duvezin-Caubet et al., 2006; Ishihara et al., 2006; Sato et al., 2011). This change in mitochondrial morphology enables cytochrome *c* to be released, which promotes apoptosis to activate proteases called caspases (Jiang and Wang, 2004; Wasilewski and Scorrano, 2009; Semenzato et al., 2011; Julien and Wells, 2017). These findings explain how aurilide triggers apoptosis through its interaction with PHB1 and suggest that aurilide results in fragmented mitochondria by inhibiting PHB1, eventually triggering apoptosis which kills cells. This apoptosis may have potential as a treatment against cancer.

### Prohibitins and Melanogenin

Melanogenin is a ligand for PHBs (Djehal et al., 2018). Knockdown of PHB1 and PHB2 with siRNA demonstrated that PHBs are necessary for induction of pigmentation by melanogenin (Snyder et al., 2005). Two melanogenin analogs, Mel41 and Mel9, activate LC3, which interacts with PHB2 (Djehal et al., 2018). These melanogenin analogs have increased microphthalmia-associated transcription factor expression. Mel41 and Mel9 promote the melanocytic production of melanin. Another melanogenin analog, Mel55, interacts with PHB1 (Djehal et al., 2018).

The involvement of PHBs in melanin production suggests that targeting PHBs may be a promising therapeutic approach for pigmentary disorders, which can be common in the elderly population (Armenta et al., 2019). Melanogenin and melanogenin analogs also have potential as treatments against cancer since they can trigger apoptosis in tumor cells, such as melanoma cells (Djehal et al., 2018). Djehal et al. (2018) also found that in addition to interacting with melanogenin, PHBs appear to modulate pigmentation in melanoma cells by interacting with fluorizoline.

### Prohibitins and Rocaglates

Rocaglates (also known as flavaglines) are from the genus *Aglaia*, which contains species that are used in traditional medicine (Ebada et al., 2011). Rocaglates interact with PHBs and have potential as a cancer treatment (Basmadjian et al., 2013). Rocaglamide is a rocaglate (Ebada et al., 2011). Rocaglamides interact directly with PHB1 and PHB2 and prevent their interaction with CRaf, leading to inhibition of the CRaf-MEK-ERK pathway (Polier et al., 2012). This inhibition suppresses malignant cell proliferation, cell growth and division, and protein synthesis. Polier et al. (2012) observed that the effect of rocaglamides on the CRaf-MEK-ERK cascade was similar to the effect of PHB1 knockdown.

Rocaglates also prevent energy production and mitophagy in cancer cells. The rocaglates FL3 and rocaglamide inhibit mitophagy by blocking the interaction between PHBs and PARL (Wang et al., 2020). These findings suggest rocaglamide could be an effective treatment against cancer through its interactions with PHBs.

### Prohibitins and Fluorizoline

Using molecules to target PHBs and induce apoptosis could be a potential treatment for cancer. Fluorizoline is a synthetic ligand for both PHB1 and PHB2 and triggers apoptosis of chronic lymphocytic leukemia cells (Pérez-Perarnau et al., 2014; Cosialls et al., 2017). Through its interaction with PHBs, fluorizoline induces apoptosis by increasing levels of NOXA protein, which is a pro-apoptotic B-cell lymphoma 2 family member (Oda et al., 2000; Cosialls et al., 2017). PHBs are also necessary for fluorizoline to trigger this apoptosis (Moncunill-Massaguer et al., 2015). In addition, cells with PHB1 depletion are resistant against cell death from fluorizoline. Fluorizoline interacts with PHBs to trigger apoptosis in acute myeloid leukemia (AML) cells (Pomares et al., 2016). Fluorizoline also triggered these AML cells to differentiate and repressed the clonogenicity of these cells.

### Rocaglamide and Fluorizoline in Cancer

Ligands for PHBs may also be potential treatments for lung cancer. PHB1 is upregulated in human non-small cell lung cancers (Jiang et al., 2013). Yurugi et al. (2017) observed that the higher the level of PHB1, the less likely the patient is to survive. This result is in line with a previous study which found that overexpressing PHB1 increased metastasis, increased mortality, and led to large cervical tumors in mice (Chiu et al., 2013).

Fluorizoline and rocaglamide stop epidermal growth factor/RAS-induced CRaf activation (Yurugi et al., 2017). This effect is a potential treatment against cancer since CRaf is necessary for tumorigenesis mediated by KRAS (Blasco et al., 2011). Rocaglamide prevents the migration and growth of lung tumor cells with mutated KRAS (Yurugi et al., 2017). Mutated KRAS often results in increased activity of ERK1/2 (Luan et al., 2014). PHB1 interacts directly with CRaf to activate ERK1/2 (Rajalingam et al., 2005). In melanoma cancer, this activation of ERK1/2 mediates resistance to the BRAF inhibitor drug vemurafenib and in melanoma cells, rocaglamide A was found to undo this resistance (Doudican and Orlow, 2017).

Rocaglamide and fluorizoline inhibit CRaf/ERK pathways by interfering with the interaction between CRaf and PHB1 (Luan et al., 2014; Yang et al., 2018). Rocaglamide was found to interfere with the interaction between PHB1 and CRaf by changing PHB1’s localization to the plasma membrane, leading to decreased RAS-ERK signaling. This decreased signaling suppresses metastasis and tumor growth (Polier et al., 2012; Luan et al., 2014). Luan et al. (2014) also found that in mice with tumors, treatment with rocaglamide lengthens lifespan.

### Prohibitins and Spiro-Oxindoles

Spiro-oxindoles and their interaction with PHBs have relevance in cardiology and cancer treatments. Doxorubicin is an anthracycline that is used as an anticancer drug (Carvalho et al., 2009). However, doxorubicin can result in cardiotoxicity (Olson and Mushlin, 1990; Singal and Iliskovic., 1998). It was found that 2′-phenylpyrrolidinyl-spirooxindole and its analogs protect against cytotoxicity from doxorubicin (Elderwish et al., 2020).

Spiro-oxindoles bind both PHB1 and PHB2 (Elderwish et al., 2020). This binding supports the survival of cardiomyocytes by leading to STAT3 phosphorylation (Elderwish et al., 2020). Rocaglates (flavaglines) have a similar cardioprotective effect. In mice treated with doxorubicin, the flavagline FL3 was found to reduce mortality by 50% (Bernard et al., 2011). Knockdown of PHB1 and PHB2 with siRNA prevented FL3’s cardioprotection and STAT3 phosphorylation (Qureshi et al., 2015). These findings suggest that targeting interactions with PHBs can help protect against cardiotoxicity in cancer treatments.

### Prohibitins and Nitric Oxide

The interactions between PHBs and small molecules could reveal the role of PHBs in neurodegenerative diseases. Upregulation of PHB1 increases neuroprotection in mice against brain damage from ischemia (Kahl et al., 2018). This finding is consistent with another study demonstrating that PHB1 is upregulated in ischemic preconditioning and decreases the number of neurons that die from injury (Zhou et al., 2012).

Brain ischemic preconditioning is an example of neuroprotection and increases the brain’s tolerance to ischemia, or reduced blood flow to the brain (Liu, X. Q. et al., 2009). Nitric oxide (NO), a neurotransmitter, regulates PHB1 and contributes to its neuroprotective effects (Qu et al., 2020). PHB1 and NO are necessary for ischemic preconditioning (Qu et al., 2020). NO directly regulates PHB1 through post-translational modification by protein S-nitrosylation (Qu et al., 2020). However, it is not known how protein S-nitrosylation of PHB1 by NO leads to neuroprotection.

### Prohibitins, Sulfonyl Amidines, and Phosphoryl Amidines

Targeting PHBs also has potential as a treatment for osteoporosis. Osteoclastogenesis can contribute to osteoporosis through differentiation of hematopoietic stem cells into osteoclasts (Boyle et al., 2003). These osteoclasts can lead to decreased bone density by resorbing bone, which can contribute to osteoporosis if there is less formation of bone than resorption of bone (Boyle et al., 2003; Harada and Rodan, 2003).

Sulfonyl amidines and phosphoryl amidines have relevance in osteoporosis because they limit resorption of bone and inhibit differentiation into osteoclasts (Lee M. Y. et al., 2010). PHB1 binds to sulfonyl amidine compounds (Chang et al., 2011). PHB1 also prevents the formation of osteoclasts (Lee et al., 2015). However, it is not known exactly how PHB1 affects sulfonyl amidines (Wang et al., 2020).

### Prohibitins and ERAP

Targeting PHB2 interactions with other molecules may be a potential treatment for breast cancer. ERα activity-regulator synthetic peptide (ERAP) disrupts the interaction between PHB2 and brefeldin A-inhibited guanine nucleotide-exchange protein 3 (BIG3) (Kim et al., 2009; Yoshimaru et al., 2013). Another molecule that directly interacts with PHB2 is xanthohumol (XN). Similar to ERAP, XN prevents the growth of breast cancer cells that are positive for ERα by disrupting the interaction between PHB2 and BIG3 (Yoshimaru et al., 2014). In most cases of breast cancer, there is BIG3 overexpression (Kim et al., 2009).

BIG3 affects the localization of PHB2. PHB2 interacts with ERα and suppresses transcription of ERα (Kim et al., 2009). This interaction occurs in the nucleus. In contrast, in breast cancer cells, PHB2 interacts with BIG3 in the cytoplasm. PHB2 is trapped in the cytoplasm due to its interaction with BIG3 (Kim et al., 2009; Yoshimaru et al., 2013). This interaction prevents PHB2 from suppressing transcription of ERα in the nucleus.

ERα has relevance in the severity of breast cancer. ERα interacts with E2 to increase metastasis and proliferation of breast cancer cells (Yager and Davidson, 2006). Using ERAP to disrupt the interaction between PHB2 and BIG3 can stop breast cancer cells that are positive for ERα from growing and make these cells more responsive to tamoxifen (Yoshimaru et al., 2013).

In addition to wild type (WT) ERα, PHB2 can bind to ERα mutants, such as D538G and Y537S, which are often present in breast cancers that are resistant to hormonal therapy (Chigira et al., 2015). As a result, treatment with ERAP could be used in breast cancer cells that are positive for ERα to undo resistance to hormonal therapies such as tamoxifen (Yoshimaru et al., 2013). This finding has importance since the majority of breast cancers that are positive for ERα become resistant to tamoxifen (Riggins et al., 2007).

A later study found that when ERAP disrupts the interaction between PHB2 and BIG3, PHB2 is able to interfere with the interaction of ER2α with a wide variety of other molecules, such as EGFR, human epidermal growth factor 2 (HER2), insulin-like growth factor 1 receptor beta (IGF-1Rβ), and PI3K (Yoshimaru et al., 2015). PHB2 repressed the proliferation of breast cancer cells that were positive for ERα by decreasing phosphorylated Akt, MAPK, and ERα, and by HER2, EGFR, and IGF-1Rβ inhibition.

### Prohibitins, CRaf, Ras, and Akt

PHB1 interacts with CRaf (Raf-1), which can activate pathways involving Ras that support cancer and its invasiveness, such as the CRaf/ERK, PI3K/Akt, and RalGEF/Ral pathways (Karnoub and Weinberg, 2008). PHB1 is necessary for Ras to activate CRaf, which is important for controlling migration and cell adhesion for epithelial cells (Rajalingam et al., 2005). Without PHB1, Raf-1 kinase and activated Ras are unable to interact. Another study expanded on this finding by revealing that phosphorylated PHB1 at T258 is important for activating CRaf, for the direct interaction between CRaf and PHB1, and for cancer cell invasiveness (Chiu et al., 2013).

PHB1 is phosphorylated at T258 by Akt and phosphorylated PHB1 interacts with Akt, Ras, and Raf-1 (Han et al., 2008; Chiu et al., 2013). In contrast, dephosphorylating PHB1 at T258 reduced tumor cell invasiveness and metastasis and decreased epithelial-mesenchymal transition (Chiu et al., 2013). Together, these findings suggest that PHB1 phosphorylated at T258 by Akt can increase the invasiveness of cancer by direct interaction with Raf-1. This interaction allows Ras to interact with and activate Raf-1 to control signaling cascades (Rajalingam et al., 2005; Han et al., 2008; Karnoub and Weinberg, 2008; Chiu et al., 2013).

### Prohibitins, Rocaglates, and Hepatitis C Virus

Older patients have a greater likelihood of developing chronic hepatitis C virus (HCV) infection (Reid et al., 2017). Interactions between small molecules and PHBs may be useful to protect against viruses. Both PHB1 and PHB2 are involved in HCV entry into human hepatocytes (Liu et al., 2015). PHB1 and PHB2 facilitate entry of HCV by associating with E2, a viral glycoprotein (Vieyres et al., 2010; Liu et al., 2015). E2, along with another glycoprotein called E1, are known to induce cells to take up the viral particle through endocytosis (Meertens et al., 2006). The use of rocaglates could be a useful protection against HCV entry (Liu et al., 2015).

Use of rocaglamide prevents the entry of HCV by preventing the interaction between PHB1, PHB2, and CRaf (Liu et al., 2015). Knockdown of PHBs does not alter HCV’s ability to bind to cells (Liu et al., 2015). However, PHB1 and PHB2 were found on the plasma membrane and were important for HCV entry after HCV binding to the cell (Liu et al., 2015). This study also found another rocaglate, aglaroxin C, which had a greater effect on preventing HCV entry than rocaglamide.

The study by Liu et al. (2015) suggests rocaglates targeting PHBs could be used to provide protection against viruses such as HCV. Similar to HCV, DENV 3, a serotype of the dengue virus (DENV), formed interactions with PHB1 and PHB2 to enter into cells (Clyde et al., 2006; Sharma et al., 2020). Other viruses, such as chikungunya virus and enterovirus 71, utilize PHB1 for entry into cells (Wintachai et al., 2012; Too et al., 2018).

### Prohibitins, Rocaglates, and Chikungunya Virus

Rocaglates (flavaglines) could also be used to protect against the chikungunya virus (CHIKV). Like HCV, CHIKV utilizes PHBs to enter cells. The E2 protein of CHIKV interacts with PHB1 to allow the virus to infect microglial cells (Wintachai et al., 2012). The flavaglines FL3 and FL23 protect cells from infection by CHIKV (Wintachai et al., 2015). Increased viral replication of CHIKV has been observed in aged Rhesus macaques, which have a weaker immune response than adult Rhesus macaques (Messaoudi et al., 2013). Rocaglates may be able to be used to counteract this age-related effect. The rocaglate derivative silvestrol (Pan et al., 2014) inhibits replication of CHIKV and inhibits eIF4A, which is an RNA helicase (Henss et al., 2018).

### Prohibitins and Coronaviruses

Older patients have a greater chance of developing life-threatening diseases from COVID-19 (Liu et al., 2020). PHBs interact with proteins of SARS-CoV-2, which is the virus responsible for COVID-19 (Andersen et al., 2020). SARS-CoV-2 has proteins called nonstructural proteins (nsps) numbered 1–16 (Chen et al., 2020). It was found that nsp2 interacts with both PHB1 and PHB2 (Cornillez-Ty et al., 2009). However, it is still not known exactly how nsp2’s interaction with PHB1 and PHB2 affects cells (Nebigil et al., 2020).

LC3 also interacts with PHBs. Coronaviruses lead to formation of double membrane vesicles with LC3-I, which enables the virus to take over intracellular membranes and to replicate (Reggiori et al., 2010). LC3 knockdown prevents cells from being infected by coronaviruses (Reggiori et al., 2010). LC3-I, PHBs, and nsp2 may interact as joint complex (Nebigil et al., 2020). This suggests that targeting PHBs could affect this joint complex and disrupt the entry of coronaviruses into cells.

### Prohibitins, JI051, and JI130

Other compounds that interact with PHBs are JI051 and JI130. JI051 and JI130 are synthesized compounds that inhibit the proliferation and growth of cancer cells (Perron et al., 2018). During adulthood, there is an association between cancer development and abnormal signaling of Hes family basic helix-loop-helix transcription factor 1 (Hes1). JI051 interacts with PHB2 (Perron et al., 2018). This interaction results in stabilization of the Hes1 and PHB2 interaction, leading to cessation of the cell cycle in the G2/M phase. In addition, JI051 inhibits the proliferation of HEK293 cells and JI130 decreases the volume of tumors. These findings suggest JI051 and JI130 could be potential treatments for cancer.

### Prohibitins and Capsaicin

Another compound that interacts with PHBs is capsaicin, which is found in hot chili peppers (Reyes-Escogido et al., 2011; Wang et al., 2020). Capsaicin can bind directly with PHB2 and result in the translocation of PHB2 from the inner mitochondrial membrane to the nucleus (Kuramori et al., 2009). In the nucleus, PHB2 increases the transcriptional activity of p53, which induces apoptosis. Capsaicin suppresses growth and triggers apoptosis in leukemic cells (Ito et al., 2004). This effect was greater for cells that were positive for p53 than for cells that were null for p53. Capsaicin also leads to mitochondrial membrane potential disruption and triggered cytochrome *c* release from the mitochondria, which is another sign of apoptosis (Kuramori et al., 2009).

### Prohibitins and Adipotide

The risk of abdominal obesity increases with age and obesity can result in insulin resistance (Jura and Kozak, 2016). Targeting PHBs has been shown to undo obesity in animal models. The peptidomimetic adipotide interacts with PHB1 in the adipose vasculature in mice (Kolonin et al., 2004). This interaction was found to combat obesity and cause weight loss in Old World monkeys, by resulting in apoptosis of the blood vessels of adipose tissue (Barnhart et al., 2011). Adipotide also resulted in decreased insulin resistance (Barnhart et al., 2011). There has not been any additional research on adipotide since 2012 (Wang et al., 2020).

## Future Directions

Much of the full function of PHBs in aging remains unknown. One of the most important questions that needs to be addressed is the number of conflicting studies about whether PHBs suppress or promote cancer and whether PHBs increase or decrease apoptosis. It would be beneficial to learn more about what factors make PHBs tumor supporters and what factors make them tumor suppressors.

More studies might be able to determine if PHBs could be used as a diagnostic tool for age-related diseases. There is potential for PHBs to be used as biomarkers for age-related problems such as oxidative stress and for diseases that increase with age such as diabetes (Lee H. et al., 2010; Supale et al., 2013; Dong et al., 2016). To further understand the role of PHBs in aging, it may be beneficial to perform observational studies on people with long health spans and lifespans to see how their expression of PHBs may differ from the general population. It would also be valuable to find out more information about the role of PHBs in Parkinson’s disease since there are only a few studies on this topic (Ferrer et al., 2007; Pickrell and Youle, 2015; Dutta et al., 2018).

An additional area to investigate is the effects of upregulation of PHBs on lifespan since many studies regarding the effect of PHBs on lifespan are only examining knockdown of PHBs (Artal-Sanz and Tavernarakis, 2010). It would also be beneficial to further examine the differences in strain genetic backgrounds that may contribute to the opposing effects on replicative lifespan in yeast (Kirchman et al., 1999). Another question deserving more attention is the relationship between PHB1 and PHB2. It should be determined why deletion of both subunits results in a greater decrease in lifespan than the deletion of only one or the other if a lack of PHB1 corresponds to a lack of PHB2 and vice versa (Berger and Yaffe, 1998). Little is known about the structure of the PHB complex, which is another area that could be studied in the future (Artal-Sanz and Tavernarakis, 2009a).

Another area of study is the relationship between apoptosis and senescence for PHBs and how it relates to cancer. It seems that induction of senescence by PHBs would be beneficial to treat cancer by preventing entry into S phase (Jupe et al., 2001). However, cells that are senescent are more resistant to apoptosis, suggesting that PHBs may also protect cancer cells from apoptosis (Marcotte et al., 2004).

There is an extensive amount of studies examining the targeting of PHBs as a potential treatment for cancer (Koushyar et al., 2015). However, there are not as many studies examining the therapeutic potential of PHBs in other diseases related to aging. For example, there are not many studies regarding the targeting of PHBs to treat neurodegenerative diseases. Nitric oxide has been found to be involved in neuroprotection, but its role in neurodegenerative diseases, as well as how its regulation of PHB1 results in neuroprotection, is not clear (Qu et al., 2020). There are also few studies that examine the therapeutic potential of PHBs in osteoporosis and how PHB1 affects sulfonyl amidines which limit bone resorption (Lee M. Y. et al., 2010; Chang et al., 2011). The therapeutic potential of PHBs in other age-related diseases such as Alzheimer’s disease, Parkinson’s disease, osteoarthritis, hypertension, diabetes, and cardiovascular diseases are all areas of research that could be expanded in the future.

## Conclusion

PHBs are involved in aging through cellular senescence, apoptosis, and oxidative stress, and have functions in age-related diseases such as Alzheimer’s disease, Parkinson’s disease, diabetes, and cancer (Ferrer et al., 2007; Artal-Sanz and Tavernarakis, 2009a; Supale et al., 2013; Lachén-Montes et al., 2017).

One particularly compelling hypothesis is that PHBs can modulate aging through mitophagy to maintain mitochondrial quality control (Youle and van der Bliek, 2012). There is also evidence that PHBs can impact cellular senescence and shift oxidative phosphorylation to anaerobic respiration (Rastogi et al., 2006a; Vessal et al., 2006).

There are conflicting studies about whether PHBs induce apoptosis or suppress it (Liu et al., 2012; Peng et al., 2015). PHBs may have a potential role as a treatment for cancer. However, more research needs to be conducted to understand what factors may make PHBs tumor promoters and what factors may make PHBs tumor suppressors.

**FIGURE 1:**
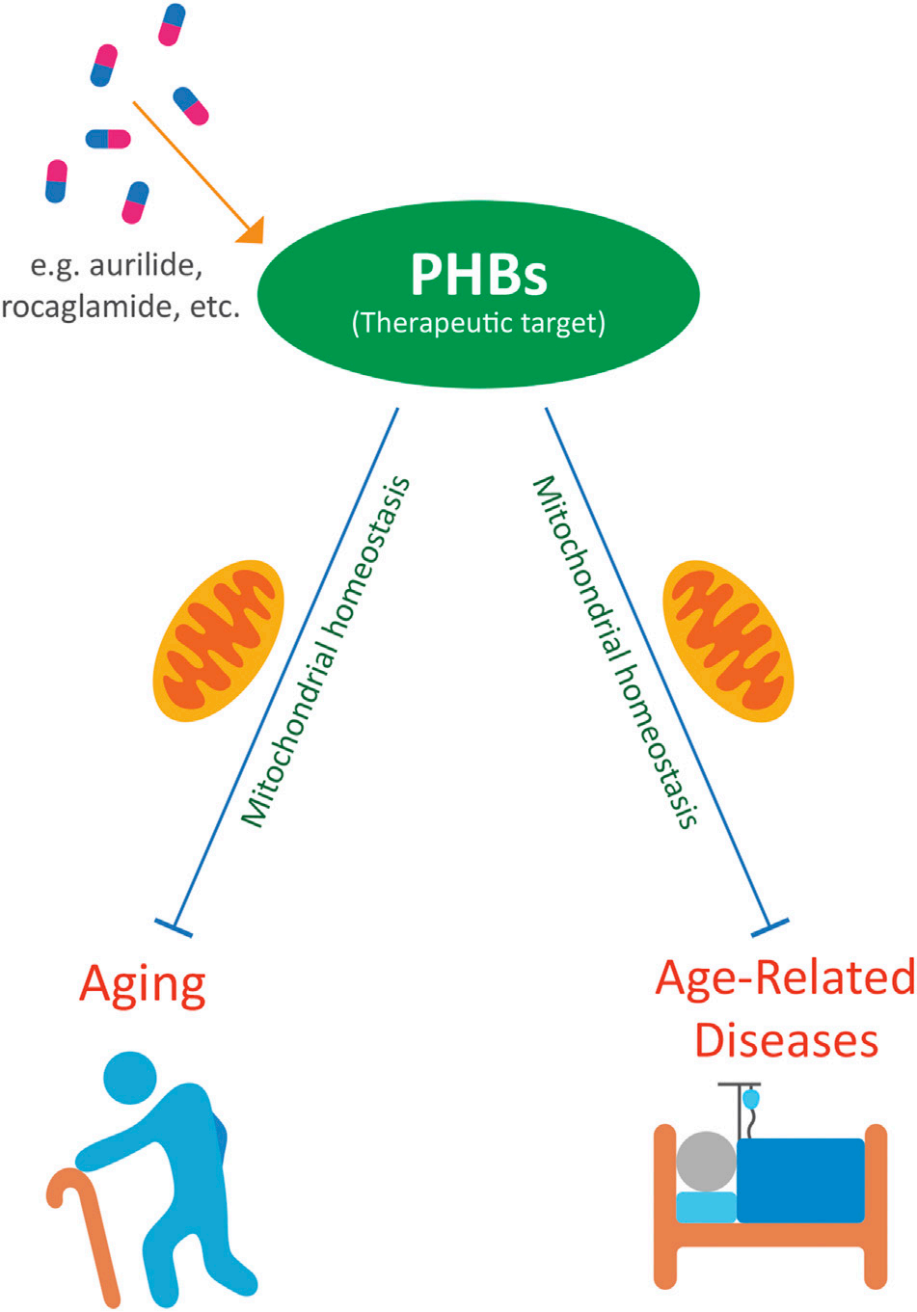
PHBs as potential therapeutic targets to ameliorate aging and age-related diseases.

PHBs have therapeutic potential in a variety of age-related diseases. Targeting PHBs with compounds such as rocaglates, aurilide, fluorizoline, melanogenin, ERAP, capsaicin, JI051, and JI130 may be potential treatments against cancer (Ito et al., 2004; Sato et al., 2011; Yoshimaru et al., 2013; Cosialls et al., 2017; Yurugi et al., 2017; Djehal et al., 2018; Perron et al., 2018). Rocaglates may also protect against viral diseases that can be more severe in the elderly (Wintachai et al., 2015; Nebigil et al., 2020). Spiro-oxindoles can protect against cardiotoxicity in cancer patients (Elderwish et al., 2020). Nitric oxide is involved in neuroprotection, which may be relevant to neurodegenerative diseases (Qu et al., 2020). Sulfonyl amidines have relevance to osteoporosis and limit bone resorption (Lee M. Y. et al., 2010; Lee et al., 2015). Targeting PHBs with these compounds may be potential treatments against a wide variety of diseases related to aging (Figure 1).
